# Regional cultures and insufficient sleep in the United States

**DOI:** 10.1186/s44167-023-00043-3

**Published:** 2024-01-04

**Authors:** Nicolaas P. Pronk, Ross Arena, Deepika Laddu, Colin Woodard

**Affiliations:** 1 HealthPartners Institute, 8170 33rd Avenue South, Bloomington, MN 55425 USA; 2 Department of Health Policy and Management, University of Minnesota, 420 Delaware St SE, Minneapolis, MN 55455 USA; 3 Healthy Living for Pandemic Event Protection (HL – PIVOT) Network, 1919 W. Taylor St. 454 AHSB, Chicago, IL 60612 USA; 4 Department of Physical Therapy, College of Applied Science, University of Illinois, Chicago, IL 60612 USA; 5 Nationhood Lab, Pell Center for International Relations and Public Policy, Salve Regina University, 100 Ochre Point Ave, Newport, RI 02840 USA

**Keywords:** Sleep, Insufficient sleep, Sleep Hygiene, Regional cultures, American Nations

## Abstract

**Background:**

Insufficient sleep can increase the risk of health problems and chronic conditions including cognitive problems, increased inflammation, diabetes and cardiovascular disease, and overall mortality. In this report, insufficient sleep, as a cultural experience, was tracked across the United States according to the American Nations model of U.S. regional cultures.

**Methods:**

County Health Rankings & Roadmaps program data relating to insufficient sleep were matched at the zip-code level with the American Nations dataset from the Nationhood Lab. Percentages for the insufficient sleep metric were then estimated using the population of each of the American Nations.

**Results:**

High levels of sleep insufficiency across all regional cultures indicate considerable room for improvement and a potential need to prioritize sleep hygiene as a health concern. ANOVA results show significant differences among the 13 American Nations and indicate variation in sleep outcomes between cultural regions. Follow-up post hoc analyses appear to support the notion that those regional cultures that place a higher value on social investments tend to report lower levels of sleep insufficiency. Furthermore, the insufficient sleep pattern noted in this report is consistent with those reported earlier for physical inactivity, obesity, and diabetes.

**Conclusions:**

Factors and circumstances occurring in the Midwest and the South-Central states appear to be responsible for the observed patterns. Future research should consider reviews of policies and related practices adopted across the American Nations to identify causal patterns.

## Introduction

Sleep has been recognized as an important health behavior associated with health outcomes. Research indicates the mean-predicted 10-year cardiovascular risk to be lowest among adults who reported sleeping 7 h per night and increased as participants reported sleeping fewer and more hours [1]. In a recent synthesis of reviews, the most favorable associations between sleep duration and health outcomes follow U-shaped dose-response curves with the lowest risk typically observed at approximately 7–8 h per day [2]. Despite the concluding recommendation of 7–8 h/day of sleep for adults, it is important to recognize an observed large inter-individual variability that may affect how the recommendations apply to a given person. National Sleep Foundation recommendations, which are evidence-based and consensus-driven [3], corroborate this U-shaped dose-response with elevated sleep insufficiency risks at < 7 h/day. Hence, insufficient sleep patterns, defined as fewer than 7 h/day of sleep, can increase risk of health problems and chronic conditions including cognitive issues, increased inflammation, diabetes, cardiovascular disease, and overall mortality [4–9].

Sleep has also been associated with cultural factors and experiences. Culture may be defined as the shared values, norms, and codes that collectively shape a group’s beliefs, attitudes, and behavior through their interaction in and with their environments [10]. As a dynamic experience, acculturation may influence health behaviors that impact sleep patterns. In a narrative review of the literature, Airhihenbuwa and colleagues argue that sleep is a cultural experience with positive, negative, and unique elements that deserve additional attention and represents a dominant aspect of life that varies among diverse groups or peoples [10]. International sleep duration comparisons reveal substantial cross-cultural variability. For example, individuals in East Asian (especially Japanese) cultures tend to sleep less than individuals in Western cultures [11]. As a result, one might expect Japan to experience higher prevalence of health risks, chronic conditions, and lower longevity than, for example, North American countries (U.S. or Canada). Yet, this is not the case, as evidenced by various indicators such as obesity, diabetes, and life expectancy [12–14]. In fact, recent evidence suggests that despite sleeping significantly less than European Canadians, Japanese Canadians slept less efficiently, yet reported being less tired and having better health. Furthermore, acculturation effects were observed as, relative to European Canadians, Japanese Canadians showed weaker associations between sleep and physical health, yet Asian Canadians’ sleep behaviors were similar to those of European Canadians [15]. Hence, despite serving fundamental biological functions, sleep behaviors may be influenced by both initial childhood culture as well as acculturation when assimilating to a new place.

Many health statistics in the U.S. are based on surveillance measures tracked at the county or state level. Yet regional cultures may blur county or state borders. Woodard used historical research techniques to track and map “first settler effects” [16] and competing North American colonization streams [17]. He identified 11 major regional cultures on the continent north of the 22nd parallel, plus two smaller enclaves of regional cultures (i.e., Spanish Caribbean and Greater Polynesia) lying largely or entirely outside this space [18]. This American Nations model has been applied to explain differences in entrepreneurship [19], economic development [20], mortality [21], gender wage gaps [22], personality characteristics [23], gun violence [18], and voting behavior [24].

An in-depth description of the regional cultures, such as delineated by Woodard [16], is beyond the scope of this report. Instead, here we present the unique principles and identities of the 13 American Nations in Table 1 including some overarching observations related to these regional cultures. Historically, people who founded the settler-colonial societies in various areas of what is now the U.S. came from different parts of Europe and brought with them different cultures with distinct religious, ethnographic, ideological, economic and political characteristics. These colonial projects competed in colonial times, even when they belonged to the same (English and, later, British) empire. As outlined in Table 1, each region had their own fundamental principles, and these were often contradictory. A nation may be defined as a group of people who share the same culture. Based on this premise, the American Nations represent a group of 11 major nations and two smaller enclaves (which we recognize in this report as well).

**Table 1:** The Identities of the ‘American Nations’

1	Yankeedom (pop. 52.6 million) Founded by Puritans who sought to perfect earthly society through social engineering, individual denial for common good, and the assimilation of outsiders. The common good – ensured by popular government - took precedence over individual liberty when the two were in conflict.
**2**	**New Netherland** (pop. 20.9 million) Dutch-founded and retains characteristics of 17th century Amsterdam: a global commercial trading culture, materialistic, multicultural, and committed to tolerance and the freedom of inquiry and conscience.
**3**	**Tidewater** (pop. 13.2 million) Founded by lesser sons of landed gentry seeking to recreate the semi-feudal manorial society of English countryside. Conservative with strong respect for authority and tradition, this culture is rapidly eroding because of its small physical size and the massive federal presence around D.C. and Hampton Roads.
**4**	**Greater Appalachia** (pop. 61.5 million) Settlers overwhelmingly from war-ravaged Northern Ireland, Northern England and Scottish Lowlands were deeply committed to personal sovereignty and intensely suspicious of external authority.
**5**	**The Midlands** (pop. 35.9 million) Founded by English Quakers, who believed in humans’ inherent goodness and welcomed people of many nations and creeds. Pluralistic and organized around the middle class; ethnic and ideological purity never a priority; government seen as an unwelcome intrusion.
**6**	**Deep South** (pop. 48.0 million) Established by English Barbadian slave lords who championed classical republicanism modeled on slave states of the ancient world, where democracy was the privilege of the few and subjugation and enslavement the natural lot of the many.
**7**	**New France** (pop. 2.6 million) An enclave of a larger culture encompassing Quebec and parts of Atlantic Canada, the legacy culture was consensus driven, tolerant, and comfortable with government involvement in the economy, though these characteristics appear to have collapsed in much of Cajun country in recent decades.
**8**	**El Norte** (pop. 34.3 million) Borderlands of Spanish-American empire, so far from Mexico City and Madrid that it developed its own characteristics: independent, self-sufficient, adaptable, and work-centered. Often sought to break away from Mexico to become independent buffer state, annexed into U.S. instead.
**9**	**Left Coast** (pop. 18.8 million) Founded by New Englanders (who came by ship) and farmers, prospectors and fur traders from the lower Midwest (by wagon), it’s a fecund hybrid of Yankee utopianism and the Appalachian emphasis on self-expression and exploration.
**10**	**Far West** (pop. 30.3 million) Extreme environment stopped eastern cultures in their path, so settlement largely controlled by distant corporations or federal government via deployment of railroads, dams, irrigation, and mines; exploited as an internal colony, with lasting resentments.
**11**	**First Nation** (pop. 61 thousand) Native American groups that generally never gave up their land by treaty and have largely retained cultural practices and knowledge that allow them to survive in this hostile region on their own terms. The nation is now reclaiming its sovereignty, having won considerable autonomy in Alaska and Nunavut and a self-governing nation state in Greenland that stands on the threshold of full independence.
12	**Greater Polynesia** (pop. 1.4 million) An enclave (in the state of Hawaii) of the massive Western Pacific cultural space settled by the great celestial navigators. Communitarian social structure. Extends thousands of miles across French Polynesia, the Cook Islands, Samoa and Tonga.
13	**Spanish Caribbean** (pop. 8.2 million) The northern tip of Spain’s maritime cultural space in the Caribbean basin, a distinct culture from El Norte with an epicenter in Havana and including today’s Puerto Rico, and Dominican Republic.

In our attempts to correlate regional cultures to health-related outcomes, we recently published the geographic distribution of unhealthy living characteristics according to the American Nations model and observed once again significant heterogeneity [25]. Characteristics described have included physical inactivity, obesity, diabetes, and adequate access to exercise opportunities, but not sleep. We hypothesize that the American Nations regional cultures will show substantial variance in sleep duration, with individualistic regions – where the neglect of public goods generates stress, poor health and socioeconomic outcomes and shortened life expectancy – exhibiting shortened sleep times. Hence, it is the purpose of this study to describe insufficient sleep patterns across 13 regional cultures that collectively make up the American Nations.

## Methods

### Data sources

The most recently available U.S. data (2020) on the prevalence of insufficient sleep were obtained from the County Health Rankings & Roadmaps (CHRR) program of the University of Wisconsin Population Health Institute (https://www.countyhealthrankings.org/) [26]. The CHRR uses the Behavioral Risk Factor Surveillance System (BRFSS) (2020: https://www.cdc.gov/brfss/index.html) from the Centers for Disease Control and Prevention (CDC) as the primary source of insufficient sleep data. The BRFSS is a state-based random digit dial telephone survey that is conducted annually in all states, the District of Columbia, and U.S. territories. Data obtained from the BRFSS are representative of each state’s total non-institutionalized population over 18 years of age and have included more than 400,000 annual respondents with landline telephones or cellphones since 2011. Data are weighted using iterative proportional fitting (also called “raking”) methods to reflect population distributions. The CDC produced county estimates using single-year BRFSS data and a multilevel modeling approach based on respondent answers and their age, sex, and race/ethnicity, combined with county-level poverty. For those counties where there were no or limited data, the modeling approach borrowed information from the entire BRFSS sample as well as Census Vintage population estimates, and parametric bootstrapping method produced standard errors and confidence intervals for those point estimates [17].

The CHRR program defines insufficient sleep as the percentage of adults who self-report that they sleep less than 7 h per night on average. The insufficient sleep data are age-adjusted. Age is a non-modifiable risk factor, and insufficient sleep is among the health behaviors that may be associated with different age groups in the population [2, 3]. The CHRR program reports an age-adjusted rate to fairly compare counties with differing age structures.

Using zip-code data, the CHRR database was linked to the American Nation dataset from the Nationhood Lab, Pell Center for International Relations and Public Policy, Salve Regina University (https://www.nationhoodlab.org/) [18]. Percentages for the insufficient sleep metric were then estimated using the population of each American Nation listed in Table 1.

### Statistical analysis

Analysis of variance (ANOVA) was used to compare differences in insufficient sleep prevalence among the American Nations. When appropriate, follow-up post hoc analyses were conducted using Bonferroni multiple comparisons to determine significant mean differences judged at the *p* < 0.05 level of significance.

### Study ethics

The HealthPartners Institute Research Subjects Protection Program determined that this study is exempt from IRB review and ongoing oversight under 45 CFR Part 46 as it involves the analysis of existing, publicly available data sets.

## Results

Table 2 presents the rank-ordered prevalence indicators of insufficient sleep among the American Nations. Greater Polynesia reports the highest prevalence of insufficient sleep (40.8%). The Deep South, Greater Appalachia, Tidewater, New Netherland, and the Spanish Caribbean report prevalence estimates of 35% or greater whereas the lowest prevalence indicators were observed for Left Coast (30.1%), Far West (32.1%), and Yankeedom (32.8%).

**Table 2:** Rank-Ordered Prevalence of Insufficient Sleep by Regional Geography Organized According to the American Nations and ANOVA with Post-Hoc Comparisons Results

*Ranking*	*American Nation*	*Prevalence (%)* Mean [SE]*	*Bonferroni* *Multiple Comparisons #*
1	Greater Polynesia	40.8 [1.4]	9,11,12,13
2	New France	37.4 [0.4]	5,8,9,10,11,12,13
3	Deep South	36.3 [0.1]	4,5,6,7,8,9,10,11,12,13
4	Spanish Caribbean	36.1 [0.5]	3,11,12,13
5	First Nation	34.9 [0.7]	3
6	Greater Appalachia	34.9 [0.1]	3,8,9,10,11,12,13
7	Tidewater	34.7 [0.3]	3,8,9,10,11,12,13
8	New Netherland	34.7 [0.5]	2,3,6,7,12,13
9	Midlands	34.1 [0.1]	1,2,3,6,7,10,11,12,13
10	El Norte	33.9 [0.2]	2,3,6,7,9,11,12,13
11	Yankeedom	32.8 [0.1]	1,2,3,4,6,7,9,10,12
12	Far West	32.1 [0.1]	1,2,3,4,6,7,8,9,10,11
13	Left Coast	30.1 [0.3]	1,2,3,4,6,7,8,9,10

Note: *ANOVA results are significant at the 0.05 level; # indicates a statistically significant difference from the American Nation(s) noted [as indicated by number in the “Ranking” column] at the p < 0.05 level

**Fig. 1:**
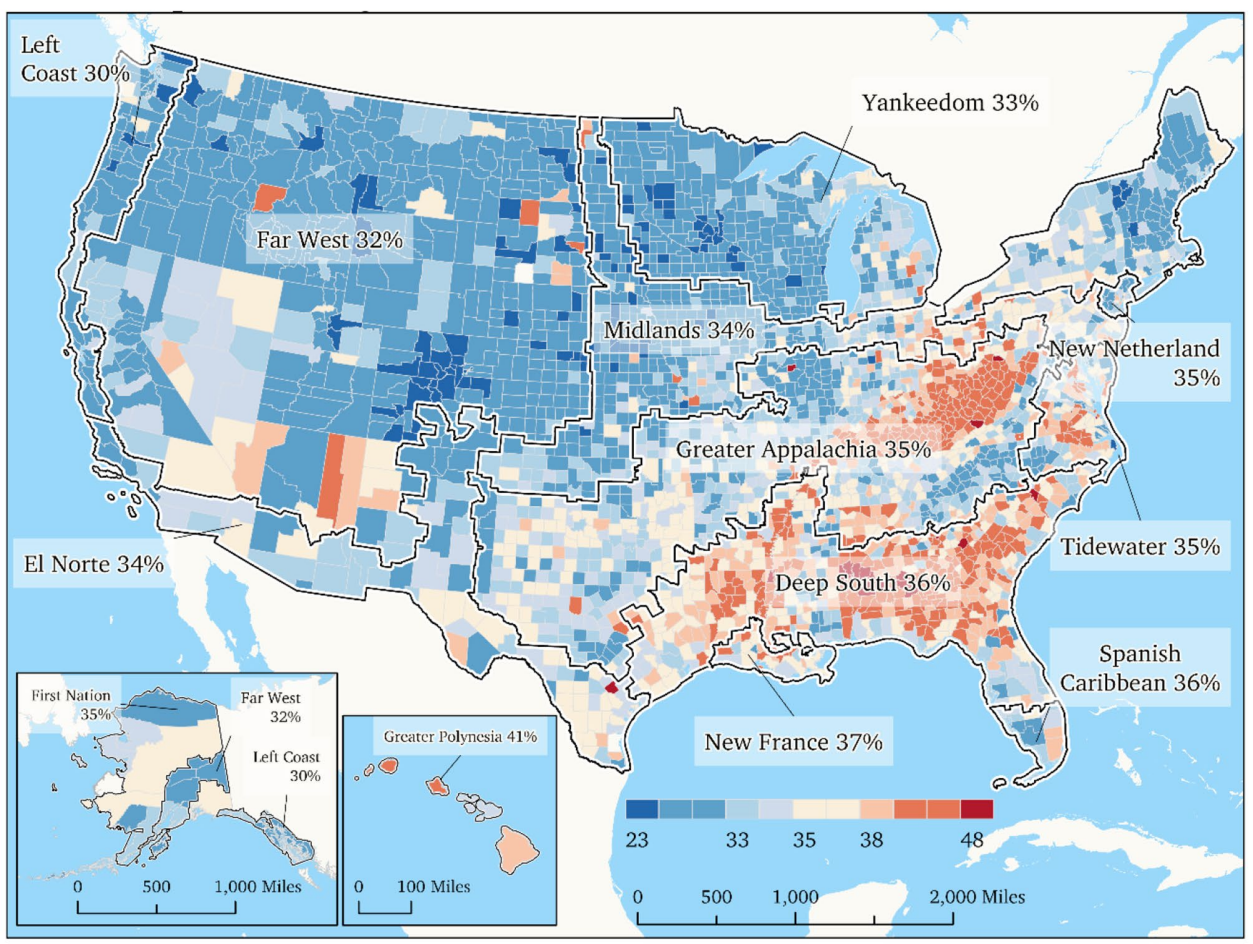
Insufficient Sleep Prevalence by Cultural Geography According to the American Nations Model. Note: John Liberty/Motivf for Nationhood Lab, 2023

Statistical differences between the 13 American Nations were observed as indicated by a significant overall ANOVA test (F = 188.083 (df = 12), MS = 1478.271, *p* < 0.001). Bonferroni post-hoc comparisons indicate statistical differences between multiple American Nations and are summarized in Table 2. These observations are supported by Fig. 1 which illustrates rounded mean data in a geographical map format in which the county-level prevalence indicators are merged according to the regional clusters that form the American Nations. This Figure clearly shows a grouping of higher sleep insufficiency regional cultures that include New France, Deep South, Greater Appalachia, Spanish Caribbean, Tidewater, and New Netherland. Higher insufficient sleep prevalence is also noted for the Hawaiian region (i.e., Greater Polynesia).

These ANOVA results indicate that differences between the 13 regional cultures that make up the American Nations show significant variability. Follow-up post hoc results indicate that regional cultures geographically positioned in the South-Eastern U.S., along the Appalachian mountain range, and the eastern seaboard south of New York report significantly higher sleep insufficiency as compared to other American Nations. Variation in sleep outcomes may indicate that regional cultures where community and social cohesion principles are supported tend to report lower levels of sleep insufficiency. However, despite significant differences among the various regional cultures, all American Nations exhibit substantial opportunity for improvement since the most favorable insufficient sleep indicator was 30.1% for Left Coast.

## Discussion

The U.S. has experienced a sustained lack of improvement in population-based healthy longevity when it is compared to other countries [27]. Part of this trend is a continued lack of improving healthy living characteristics, such as physical activity, obesity, and nutrition-related factors. Sleep is another health-related behavior that has emerged as an important variable in health-related outcomes and overall longevity [1–14]. The data presented herein indicate that room for improvement of insufficient sleep exists across all regional cultures identified in the American Nations model and that some regions—such as those that include Greater Polynesia, New France, the Deep South, or Spanish Caribbean—may need to consider prioritization of sleep hygiene as a health concern. Furthermore, the insufficient sleep trend noted in this report is consistent with those reported earlier for physical inactivity, obesity, and diabetes [25]. Factors and circumstances centered around the Midwest and the South-Central states appear to be responsible for the observed patterns, an observation corroborated by data presented on life expectancy trends over the past century [27].

These observations are corroborated by earlier reports including an analysis at the county-level by Grandner and colleagues [28] on the geographic distribution of insufficient sleep across the U.S. Insufficient sleep hotspots were found in 84 counties spread across 12 states including Alabama, Arkansas, Georgia, Illinois, Kentucky, Louisiana, Missouri, Ohio, Tennessee, Texas, Virginia, and West Virginia. Collectively, these states are represented within the American Nations’ regional cultures of the Deep South, Midlands, Greater Appalachia, Tidewater, and a single hotspot county within El Norte. The current investigation extends these findings beyond geographic variability into the realm of regional cultures. Knowledge of an underlying set of values and principles of a specific geographic regions may aid in the development of public health campaigns. As such, this report presents novel data that, in combination with county and state-level data on health, policy, and socioeconomic contexts, regional cultural differences may be useful and important in crafting powerful public health campaigns.

Moving beyond a general description of the American Nations, we highlight Yankeedom and the Deep South as two examples of distinctly different regional cultures. Yankeedom was founded by Calvinists who settled the Massachusetts Bay colony and believed they had been chosen by God to bring into being about a more perfect society that would be a “light on a hill” to guide humanity. To fulfill their mission as a covenanted people, the Puritans emphasized education, local political control, the interests of the community (over that of individuals), and the establishment of strong institutions to guide their shared “errand in the wilderness.” It is a culture that imbued strong support and faith in government and other shared institutions understood to be extensions of the community and primary tools for the furtherment of its health and goals. Like the other regional cultures, Yankeedom spread across its own nearly exclusive settlement domain, absorbing the “Old Colony” of the Pilgrims and the rest of New England in mid-1600s, much of upstate New York starting in the 1670s, and on into northern Pennsylvania, the (Connecticut-claimed) Western Reserve of Ohio, and what is now Michigan, Wisconsin, and Minnesota in the 18th and early 19th centuries.

By contrast, the Deep South was founded by English slave lords from Barbados who transplanted their fully-formed West Indies stye slave plantation culture to the subtropical lowlands of a new colony on the North American mainland that was for a time called “Carolina in the West Indies.” This culture – which eventually spread across the majority of South Carolina, Georgia, Alabama, Mississippi and Florida and parts of North Carolina, Arkansas, Tennessee, and Texas – was organized to benefit the planter oligarchy, which shunned investments in public goods and taxes or regulations affecting land and capital. Ideologically they embraced the republics of classical Antiquity, where democracy was the privilege (or “liberty”) of the few and subjugation and slavery the natural and necessary lot of the many. In the Antebellum period their leaders denounced the assertion of natural human equality in the Declaration of Independence and argued that the U.S. should be understood as a collection of Anglo-Saxon ethnostates protected by a federal umbrella. When their race-based slave system was destroyed in the 1860s they created an apartheid regime backed by legal and extralegal violence that endured for another century and into the living memory of millions of the region’s residents today.

These two regions – the superpowers of the intra-regional political struggle that has formed the backdrop for so much of U.S. history since the 1830s – represent opposing preferences regarding social policy. Yankeedom is communitarian, relatively comfortable with strong government, working through institutions, the regulation of economic and social actors, and levying taxes to pay for stronger social services, public institutions, and social safety net protections. The Deep South is individualistic, seeking to maximize the economic and political autonomy of the region’s “winners” by keeping taxation, regulations, social services, welfare provisions and the government weak. Much as Putnam [29] found in examining differences between regions in northern and southern Italy, these “American Nations” strongly differ in levels of social and civic trust and also in health outcomes, including per capita COVID-19 deaths [30], gun deaths [31], and life expectancy [32]. Greater Appalachia and, to a lesser extent, the Far West, are also individualistic sides of the spectrum (though for different reasons); New Netherland and Left Coast are reliably communitarian.

As there is “no evidence to indicate that, at the population level, the need for sleep differs across ethnicity or countries,” prominent researchers have said they believe “that a significant portion of the variation in sleep patterns seen across countries is likely to be cultural in origin” [33]. County-level sleep deprivation hotspots in the U.S. have previously been shown to have higher proportions of “younger individuals of lower socioeconomic status and poorer health” and also to have high rates of obesity, diabetes, cardiovascular and lung diseases and several types of cancer” [28]. Our results in this present study show substantial variation in sleep duration, with individualistic regions – where the neglect of public goods generates stress, poor health and socioeconomic outcomes and shortened life expectancy – exhibiting shortened sleep times.

Furthermore, we previously documented unhealthy living characteristics, including sleep patterns, across the U.S. as part of an effort to describe the syndemic relationship between unhealthy living behaviors, chronic diseases, and coronavirus disease 2019 (COVID-19) [34]. We presented data at the county or state level across the U.S. for COVID-19 death rates, physical inactivity, cigarette use, obesity, nutrition-related variables, sleep, and heart disease death rates and noted significant heterogeneity among the prevalence estimates. Interventions designed to increase the likelihood of adopting healthier behaviors may benefit from messaging and communications that align with the cultural predisposition of the population. When regional cultures blur state and county lines, an urgent need may exist to (re-)conceptualize and (re)design messaging and health promoting interventions according to regions where cultural disposition is similar.

The map presented in this brief report provides a novel approach to demonstrating the geographical considerations of insufficient sleep as a population and public health concern in the context of a cultural paradigm. Mental health, stress and anxiety have been recognized as strong factors for sleep apnea or sleep insufficiency [2, 3]. Data from the CHRR indicates that median household income, cost of living, and long commuting time are among the main indicators of poor sleep—observations that apply to areas within Greater Polynesia (such as in Honolulu County which is one of the least-rested counties that also reports long work commuting times due to multiple jobs needed to support households and a 3–6 hours’ time lag behind the rest of the U.S.). Obesity is another factor associated with sleep apnea [2, 3] and our previous research [25] has documented high obesity prevalence in similar county-level hotspots as noted by Grandner and colleagues [28]. Mental health concerns are related to social cohesion and feelings of belonging and such factors may find stronger support in communities that have strong cultural drivers that include strength of community (e.g., Yankeedom), tolerance (e.g., New Netherland), or support for social reform (e.g., the Left Coast) as compared to regional cultures that rely on individualism (e.g., the Deep South) or reject the idea of social reform (e.g., Greater Appalachia).

More specific and targeted efforts to tailor messages to specific regions across the country may result in stronger responses to calls for action. This type of approach may also spur more innovation in intervention designs that result in higher uptake of programs and increased likelihood of success in achieving projected results. Underlying cultural differences between the American Nations may also be related to the type of policies adopted by the counties and states that make up these American Nations. Future research should recognize the complexity of these interrelated issues and delving into reviews of such policies and related practices may be well-justified from a public health perspective.

### Strengths, limitations, and conclusions

The current analysis has several strengths that should be balanced against several limitations. Certainly, the results are limited by the fact that sleep insufficiency data comes from the BRFSS and is based on self-report. However, the BRFSS is a long-standing data source for population health behavior trends and has been tested for validity and reliability [35, 36]. Another limitation reflects the nature of the clusters of county-level data that make up the American Nations. Each county has an estimated rate, and some county “hot spots” rates may be dissipated by other counties within the same American Nation. As such, differences between the 13 American Nations reflect averages across the clusters of counties that define the regions. Thus, specificity may have been limited in the analyses of county clusters reported here when compared to variation between individual counties (e.g., as compared to Grandner et al. [28]). However, this limitation should be balanced against the novel approach that the clustering provides as it pulls cultural influences together, thereby allowing for an innovative perspective on the influence of regional cultures on sleep behavior.

We have presented a point of view on insufficient sleep and its association with regional cultures as defined by the American Nations model [16] which applies the well-accepted anthropological framework of “fist settler effects” [17]. As far as we know, this is the first study to report on the influence of the American Nations regional cultures on sleep insufficiency. Cultural aspects of sleep should be considered alongside and integrated with its relationships to biological, physiological, social, economic, and political considerations. The interplay between insufficient sleep, other health-related behaviors, disease outcomes, and a host of cultural, economic, social, and physical concerns needs to recognize the complexity of this dynamic situation. Therefore, we hope that additional research will address questions that place health-related behaviors such as sleep, in context of cultural considerations and call for a systems science approach to address the complexity of these challenges [32, 33, 37, 38]. Such approaches may also provide opportunities for increased use of dialogue and open debate in meeting the cultural challenges of our time and allow for innovative interventional designs that align with regional cultural dispositions of the population.

## Data Availability

The datasets generated and/or analyzed during the current study are available from the County Health Rankings & Roadmaps program at the University of Wisconsin Population Health Institute (https://www.countyhealthrankings.org/) and the Behavioral Risk Factor Surveillance System (2020: https://www.cdc.gov/brfss/index.html) from the Centers for Disease Control and Prevention. The American Nations dataset from the Nationhood Lab, Pell Center for International Relations and Public Policy, Salve Regina University (https://www.nationhoodlab.org/) are available from the upon reasonable request from Mr. Colin Woodard.

## Abbreviations

U.S.: United States
CHRR: County Health Rankings & Roadmaps
BRFSS: Behavioral Risk Factor Surveillance System
COVID-19: Coronavirus disease 2019
